# A Novel Composite Hydrogel Composed of Formic Acid-Decellularized Pepsin-Soluble Extracellular Matrix Hydrogel and Sacchachitin Hydrogel as Wound Dressing to Synergistically Accelerate Diabetic Wound Healing

**DOI:** 10.3390/pharmaceutics12060538

**Published:** 2020-06-11

**Authors:** Chien-Ming Hsieh, Weu Wang, Ying-Hsuan Chen, Pu-Sheng Wei, Yu-Hsuan Liu, Ming-Thau Sheu, Hsiu-O Ho

**Affiliations:** 1 School of Pharmacy, College of Pharmacy, Taipei Medical University, 250 Wu-Hsing Street, Taipei 11031, Taiwan; cmhsieh@tmu.edu.tw (C.-M.H.); m301106017@tmu.edu.tw (Y.-H.C.); d301106004@tmu.edu.tw (P.-S.W.); d301108012@tmu.edu.tw (Y.-H.L.); 2 Department of Surgery, School of Medicine, College of Medicine, Taipei Medical University, 250 Wu-Hsing Street, Taipei 11031, Taiwan; wangweu@tmu.edu.tw; 3 Division of General Surgery, Department of Surgery, Taipei Medical University Hospital, 252 Wu-Hsing Street, Taipei 11031, Taiwan

**Keywords:** acellular extracellular matrix, porcine skin, sacchachitin, wound dressing, hydrogel

## Abstract

Extracellular matrix (ECM) hydrogel can create a favorable regenerative microenvironment and act as a promising dressing for accelerating the healing of diabetic wound. In this study, a simple and effective decellularization technique was developed and optimized to obtain acellular extracellular matrix (*a*ECM) from porcine skin. It was found that decellularization at 30% formic acid for 72 h effectively decellularized porcine skin while retaining >75% collagen and ~37% GAG in the *a*ECM with no presence of nuclei of cellular remnants. *a*ECM hydrogel was fabricated by digesting *a*ECM with pepsin in various acidic solutions (0.1 N HCl, glycolic acid (GA) and 2-pyrrolidone-5-carboxylic acid (PCA)) and then treated with a pH-controlled neutralization and temperature-controlled gelation procedure. Based on physical characterizations, including SDS-PAGE, rheological analysis and SEM analysis, *a*ECM_HCl_ hydrogels fabricated at 25 mg/mL in 0.1 N HCl were selected. Four polymeric ECM-mimic hydrogels, including sacchachitin (SC), hyaluronic acid (HA) and chitosan (CS) and three composite hydrogels of combining SC either with *a*ECM_HCl,25_ (*a*ECM_HCl_/SC), HA (HA/SC) or CS (SC/CS) were prepared and evaluated for WS-1 cell viability and wound-healing effectiveness. Cell viability study confirmed that no hydrogel dressings possessed any toxicity at all concentrations examined and ECM_HCl_, HA and ECM_HCl_/SC at higher concentrations (>0.05%) induced statistically significant proliferation. Diabetic wound healing study and histological examinations revealed that ECM_HCl_/SC hydrogel was observed to synergistically accelerate wound healing and ultimately stimulated the growth of hair follicles and sweat glands in the healing wound indicating the wound had healed as functional tissues. The results support the great potential of this newly produced ECM_HCl_/SC composite hydrogel for healing and regeneration of diabetic wounds.

## 1. Introduction

Extracellular matrix (ECM) is the largest component of normal skin and ECM synthesis is crucial for wound healing [1]. ECM components include collagen, proteoglycans, such as glycosaminoglycan (GAG), hyaluronic acid (HA) and so forth. [2]. ECM lacks immunogenicity and its biological properties are superior to other types of dressings. Since that, many studies have demonstrated that ECM can act as a dressing creating a favorable regenerative microenvironment for repairing wounds [3]. Nevertheless, it is difficult to construct a wound dressing with the same composition as that of in vivo ECM. Consequently, decellularization of biological tissues has become an attractive strategy for obtaining ECM. The requirements for ECM after decellularization are an acceptable amount of residual DNA (<50 ng DNA per mg dry weight), preservation of the collagen component (about 75%) and retention of the ECM structure [4]. To improve the efficiency of decellularization, enzymatic, chemical and physical methods were developed for different types of tissue and species of origin [5,6,7]. Enzymatic methods for decellularization usually use proteases and chelating agents (such as ethylenediaminetetraacetic acid (EDTA)). The methods can effectively remove cellular materials and preserve the collagen components but they might disrupt the ECM structure [8], a length incubation was necessitated [9] or with impractical device to assist [10]. Chemical methods by acidic and alkaline treatments can solubilize the cellular cytoplasmic components, remove nucleic acids (for instance, DNA), preserve the structure and function of the native ECM and simultaneously disinfect the material through entering microorganisms and oxidizing microbial enzymes [11]. A simple formic acid treatment able to remove most of the cellular contents and preserve the highest ECM contents in the decellularized porcine menisci was reported by Chen et al. [12]. Physical methods can be used to decellularize tissues by disrupting cellular membranes but cellular debris cannot be removed. Therefore, physical methods are usually combined with chemical or enzymatic treatment [13,14]. Overall, it was inevitable to develop simple, effective and optimized decellularization techniques to obtain ECM from porcine skin for wound-healing medical applications.

The “wet wound healing theory” proposes that the wet healing environment is favorable to the granulation growth and to the enablement of the skin cell division, thereby promoting the complete wound healing [15]. Based on this theory, intrinsic characteristics of hydrogels or pastes are expectable to be advantageous for the wound healing process since they provide the dressing with desirable essential properties—better biocompatibility; protection from physical injury; optimal gaseous exchange and nutrients supply to cells due to high sponginess; additionally, their swellability allows for the absorption of excess exudates as well as the maintenance of an optimal moisture environment around the wound, which exhibited to accelerate the wound re-epithelialization process [16]. Hydrogels or pastes based on the natural polymers chitosan [17] and hyaluronic acid [18], an interpenetrated polymer network (IPN) composed of chitosan/oxidized hyaluronic acid/catechol terpolymer/Fe [16], pepsin-soluble collagen isolated from Nile Tilapia skin [15], micronized ADM [19], have been explored for this purpose and demonstrated to provide an extracellular matrix (ECM) mimicking microenvironment with high cell affinity and bioactive functionalities that could enhance the tissue regeneration process and has a great potential for its application as wound dressing.

Furthermore, ECM hydrogels have also been created from numerous tissue sources in almost every organ system and used to facilitate functional and constructive tissue remodeling in a variety of clinical applications, such as ischemic injuries and organ regeneration or replacement [20]. There are two key steps in the fabrication of ECM hydrogels from acellular ECM (*a*ECM)—solubilization of *a*ECM and temperature- and/or pH-controlled neutralization [21]. The most popular method to solubilize *a*ECM is to digest it in pepsin with acetic acid or dilute hydrochloric acid for different times [22,23]. The type of acidic solution and digestion time used are tailored to produce different bioactive properties for each clinical application. Then pepsin-soluble *a*ECM is treated with pH-controlled neutralization and temperature-controlled gelation procedures and manipulated to form *a*ECM hydrogels [24,25]. Furthermore, it has been reported by Guo et al. that *a*ECM may accelerate the healing velocity of uninfected, non-ischemic, full-thickness diabetic foot ulcer with showing superiority and generating no more complications compared with standard of care alone [26]. Since there are abundant porcine skin resources, it was thought that *a*ECM as a hydrogel form might be fabricated from acellular ECM obtained by decellularizing porcine skin for utilization as wound dressing to enhance the healing of diabetic wounds. In this study, therefore, a simple and effective method to decellularize porcine skin with formic acid treatment followed that reported by our labs [12] was developed and optimized, which was then further processed to produce *a*ECM hydrogels by pepsin digestion and pH-controlled neutralization and temperature-controlled gelation procedures. The extent of decellularization and major ECM components (collagen and glycosaminoglycan) remained were characterized. Moreover, the sol-gel transition was measured and a rheological analysis of the obtained *a*ECM hydrogels was conducted. In addition, hydrogels based on the natural polymers sacchachitin (SC) [27], chitosan (CS) [17] and hyaluronic acid (HA) [18] that able to provide an extracellular matrix (ECM) mimicking microenvironment and their composite hydrogels were included for comparison in in vitro cell viability of WS-1 human skin fibroblasts and the wound healing effectiveness on diabetic wound model of rat.

## 2. Materials and Methods

Acetone was purchased from ECHO Chemical (Miaoli, Taiwan). Sodium chloride (NaCl) and sodium hydroxide (NaOH) were obtained from Showa Chemical (Tokyo, Japan). Citric acid, sodium citrate, trypsin, pepsin, formic acid, 10% formaldehyde, glycolic acid, 2-pyrrolidone-5-carboxylic acid (PCA), papain, l-Cysteine hydrochloride, sodium EDTA, Trizma^®^ base, sodium dodecyl sulfate (SDS), glycerol, 2-mercaptoethanol, bromophenol blue, methanol, acetic acid, coomassie blue R-250, chitosan (CS, with a molecular weight of 50–190 kD and 75–85% extent of deacetylation) and nicotinamide (Vit.B3) were purchased from Sigma-Aldrich (St. Louis, MO, USA). Hydroxyproline kit and glycosaminoglycan (GAG) kit were supplied by Chondrex (Redmond, WA, USA). Phosphate buffered saline (PBS) and penicillin streptomycin (PS) were purchased from Corning (Corning, NY, USA). Hydrochloric acid (HCl) and triethanolamine (TEA) were obtained from Merck (Darmstadt, Germany). Protein marker was purchased from BioTools (New Taipei, Taiwan) and 30% acrylamide/bis solution (37.5:1), tetramethylethylenediamine (TEMED), ammonium persulfate (APS) and glycine were obtained from Bio-Rad (Hercules, CA, USA). Porcine type I collagen standard-FlexiCol^®^ was purchased from advanced BioMatrix (San Diego, CA, USA). Hydroxyethyl Cellulose (HEC, Natrosol 250 HHX Pharm) was supplied by Ashland LLC (Covington, KY, USA). Hyaluronic acid (HA)-Freda^®^ was obtained from Bloomage Freda Biopharm (Jinan, China). Sacchachitin (SC, 2% *w*/*v* hydrogel obtained by mechanical disintegration of sacchachitin microfiber in ddH_2_O as that reported previously [27]). Minimum essential medium (MEM), fetal bovine serum (FBS) and a picoGreen quantification kit were purchased from Thermo Fisher Scientific (Waltham, MA, USA). WST-1 cell proliferation reagent was obtained from Roche Diagnostics GmBH (Mannheim, Germany). Streptozotocin (STZ) was purchased from ChemCruz^®^ (Santa Cruz, CA, USA). Anti-CD31 antibody was obtained from Abcam (Cambridge, UK).

### 2.1. Development of a Decellularization Method for Porcine Skin

Porcine skin was obtained from a slaughterhouse in Taiwan and washed with water. Its outer and inner surfaces were scraped to remove hair and subcutaneous fat and then the skin was cut into pieces (7 × 5 cm^2^). In order to remove all of the fat, these pieces were soaked in acetone for 72 h. Then, the acetone was decanted and the pieces were cleaned with deionized water and subsequently treated with 10% sodium chloride at 4 °C for 24 h. The sodium chloride solution was decanted and the pieces were washed again with deionized water. Salt-free pieces of tissue were soaked in 1.92% citrate buffer (pH 4.3) at 25 °C for 48 h [28,29]. The swollen tissues obtained after soaking in citrate buffer were immersed for 18 h in a 0.25% trypsin solution at 25 °C. Then, they were repeatedly washed with phosphate buffer and cut into small pieces (0.5 × 0.2 cm). These small pieces were agitated at 120 rpm with different concentrations (10%, 20% and 30%) of formic acid for different times (24, 48 and 72 h). Finally, these decellularized skin were washed thorough with phosphate buffer until neutral and then lyophilized to obtain *a*ECM samples. They were stored in a desiccator until use.

### 2.2. Evaluation of the Decellularization Efficiency

#### 2.2.1. Histological Analysis

First, the decellularization efficiencies of aECM treated with different formic acid concentrations (10%, 20% and 30%) for different times (24, 48 and 72 h) were verified by histological staining. Specimens were fixed in 10% (*v*/*v*) neutral buffered formalin for 18 h and embedded in paraffin. Untreated porcine skin served as a control group. These specimens were cut into 5~10-µm thicknesses and stained with hematoxylin and eosin (H&E) to inspect fibroblast cells (stained bluish-purple by hematoxylin) and collagen fibers (stained pink by eosin) under an Olympus BX43 stereomicroscope (Olympus, Tokyo, Japan).

#### 2.2.2. Biochemical Analysis

To measure amounts of DNA, GAG and total collagen remaining after decellularization, *a*ECM samples were first digested with a papain solution at 60 °C for 16 h. Quantitation of DNA was carried out using a PicoGreen DNA detection kit (Thermo Fisher Scientific, Waltham, MA, USA). The fluorescence intensity was measured with a Cytation™ 3 cell imaging multi-mode reader (Bio-Tek, Winooski, VT, USA) at an excitation wavelength of 480 nm and emission at 520 nm. The fluorescence of the DNA-free blank was subtracted from that of the experimental groups to account for the fluorescence of the sample alone. In general, hydroxyproline was used to determine the collagen content. Amounts of hydroxyproline and GAG were respectively determined with a hydroxyproline assay kit (# 6017) and GAG kit (# 6022; Chondrex, Redmond, WA, USA) according to the manufacturer’s instructions. The absorbance against a background control was detected at respective wavelengths of 525 and 530 nm with a Cytation™ 3 cell imaging multi-mode reader. Then, the hydroxyproline content was converted to that of total collagen using a mass ratio of 7.41.

### 2.3. Preparation and Process Optimization of aECM Hydrogels

According to the criteria of decellularization efficiency (acceptable amounts of residual DNA after decellularization of <50 ng DNA per mg dry weight), an optimized *a*ECM was selected from the above experiment for use in the following hydrogel preparation and process optimization. Different weights of optimized *a*ECM (at either 10, 25 or 50 mg/mL) were digested with a solution of 1 mg/mL porcine pepsin in different acidic solutions (0.1 N HCl, glycolic acid (GA) and 2-pyrrolidone-5-carboxylic acid (PCA), designated as *a*ECM_HCl_, *a*ECM_GA_ and *a*ECM_PCA_, respectively) under a constant stirring rate (400 rpm) for 48 h at 25 °C. The acronym of these samples is listed in Table 1. Subsequently, gelation was induced by neutralizing the pH value to 7 with 2 N NaOH and heating to 37 °C. After that, the *a*ECM hydrogels were formed and were characterized.

### 2.4. Characterization of aECM Hydrogels

#### 2.4.1. Sol-Gel Phase Transition

Effects of different concentrations of *a*ECM digested with the same pepsin concentration in different acidic solution on the sol-gel status of hydrogels were evaluated using test tube inversion in a 15-mL test tube at 25 and 37 °C followed the same procedure as previously reported [30].

#### 2.4.2. Rheological Studies of *a*ECM Hydrogels

Since it was found that *a*ECM fabricated at 25 mg/mL in three acidic solutions examined was able to transform to gel or gel-like, rheological characteristics of *a*ECM_HCl,25_, *a*ECM_GA,25_ and *a*ECM_PCA,25_ hydrogels were examined with a rheometer (HAAKE™ Rotation Rheometer RS-1; Thermo Fisher Scientific, Waltham, MA, USA) using the oscillatory shear stress method followed the same procedure as previously reported [29]. The linear viscoelastic region (LVR) of a hydrogel was determined and the storage modulus (G′) and loss modulus (G″) values were compared under the same physical conditions while holding the frequency (1 Hz) constant and increasing the stress strain from 0.1 to 100 Pa and holding the temperature at either 25 or 37 °C. The rheological behavior of the *a*ECM_HCl,25_+SC hydrogel dressing was evaluated as well using the same method as described above.

#### 2.4.3. Scanning Electron Microscopy (SEM)

For SEM (Hitachi, SU3500, Tokyo, Japan), freeze-dried *a*ECM_HCl,25_, *a*ECM_GA,25_, *a*ECM_PCA,25 and_ *a*ECM_HCl,25_+SC sponges fabricated at 25 mg/mL were mounted with conductive carbon tape, sputter-coated with gold (Hitachi IB-2) and imaged on a Hitachi SU3500 SEM (Tokyo, Japan) at a 5-mm working distance and an accelerating voltage of 2.5 kV.

#### 2.4.4. Qualitative Analysis of Collagen in *a*ECM Hydrogels

For a qualitative analysis of collagen in *a*ECM hydrogels, sodium dodecylsulfate (SDS)-polyacrylamide gel electrophoresis (PAGE) was carried out, using 5% (*w*/*v*) polyacrylamide (Bio-Rad, Hercules, CA, USA) for the separating gel and 4% (*w*/*v*) polyacrylamide for the stacking gel. The *a*ECM_HCl,25_, *a*ECM_GA,25_ and *a*ECM_PCA,25_ hydrogels and a standard (porcine type I collagen, FlexiCol^®^) were prepared in 0.5 M Tris-HCl buffer (pH 6.8) containing 10% SDS, 30% glycerol, 1% 2-mercaptoethanol and 0.02% bromophenol blue and then heated to 95 °C for 5 min. Next, 8 µL of a protein marker (BioTools, New Taipei City, Taiwan), 30 µL of the standard and 30 µL of the three hydrogels were loaded and electrophoresed at 85~100 V on vertical slab gels until the bromophenol blue had moved out of the gel. Polyacrylamide gels were stained for 2 h with 0.1% Coomassie blue R-250 in acetic acid/methanol/water 2:5:5 (*v*/*v*/*v*) and were destained in 7.5% acetic acid/15% methanol.

### 2.5. Fabrication and In Vitro Cell Viability Test of Hydrogel Dressings

There listed formulations for five kinds of hydrogel dressings and three kinds of composite hydrogels in Table 2 that was subjected to evaluate the effectiveness for wound healing. Five kinds of hydrogel dressings including HEC, CS, SC, HA and *a*ECM_HCl,25_ were fabricated simply by dissolving the indicated amount in the designated solvent and three kinds of composite hydrogel including *a*ECM_HCl,25_/SC, HA/SC and CS/SC were prepared simply by mixing two hydrogel components together. These hydrogel dressings were prepared and then subjected to UV radiation for sterilization.

WS1 human skin fibroblasts (American Type Culture Collection, Manassas, VA, USA) were maintained in α-minimum essential media (MEM) (Thermo Fisher Scientific, Waltham, MA, USA) with 10% fetal bovine serum (FBS, Thermo Fisher Scientific) and 1% penicillin-streptomycin (Level Biotechnology, New Taipei City, Taiwan) at 37 °C with 5% CO_2_. Cells were seeded at a density of 10^4^ cells/well in 96-well plates. Different concentrations (0.005%, 0.01%, 0.05%, 0.1% and 0.5% *w*/*v*) of eight kinds of sterilized hydrogel dressings were added to each well. After 72 h of incubation, 10 µL of WST-1 reagent was added to each well and incubation continued at 37 °C with 5% CO_2_ for additional 3 h. Cell viability was measured using the WST-1 cell proliferation reagent (Roche Diagnostics, Mannheim, Germany). In principle, the tetrazolium salt WST-1 (4-[3-(4-Iodophenyl)-2-(4-nitro-phenyl)-2H-5-tetrazolio]-1,3-benzene disulfonate) was cleaved to a soluble formazan by mitochondrial dehydrogenase in viable cells. The proliferation of viable cells leads to an increased overall activity of these enzymes. Corresponding changes in absorbance of the dye solution were detected at a wavelength 440 nm with a Cytation™ 3 cell imaging multi-mode reader.

### 2.6. Wound-Healing Studies

#### 2.6.1. Diabetic Rats in Wound-Healing Studies

All animal experiments were performed in specific pathogen-free conditions with a protocol (no. LAC-2017-0352, 2017/12/08–2020/12/07) approved by the Laboratory Animal Center of Taipei Medical University (Taipei, Taiwan). Animal study design, requirements of laboratory animals and selection of types and number of animals have also been justified with adhering to the 3Rs principles (replacement, reduction and refinement). Sprague-Dawley rats (males, 8 weeks old) were obtained from BioLASCO Taiwan (Taipei, Taiwan) and housed under a 12-h light/dark cycle, allowed food and water ad libitum and acclimatized for 1 week before the experiment. Diabetes was induced in these rats by an intravenous (i.v.) injection of a solution of 65 mg/kg streptozocin (Santa Cruz Biochemicals, Santa Cruz, CA, USA) [31]. Blood glucose values were measured with a Roche^®^ ACCU-CHEK^®^ Performa blood-glucose meter (Roche^®^, Taipei, Taiwan) and weights were also recorded. After 7 days, if blood glucose values were >200 mg/dL, rats were considered to be diabetic. The diabetic rats were anesthetized with Zoleti^®^ (VIRBAC, Carros, France) and were used to study the wound-healing rate. The dorsal hair of a rat was removed with an electric razor. Four wounds of equal area were created with an 8-mm biopsy punch and silicon splints were put in place to prevent wound deformation and contraction. Each wound was randomly covered with an equal-sized hydrogel dressing, including one control (HEC), three hydrogels (SC, HA and CS) based on the natural polymers of sacchachitin (SC), chitosan (CS) and hyaluronic acid (HA) that are able to provide an extracellular matrix (ECM) mimicking microenvironment, *a*ECM_HCl,25_ hydrogel dressings and three kinds of composite hydrogels (*a*ECM_HCl,25_/SC, HA/SC and CS/SC). Hydrogel dressings were applied on days 0, 2, 4, 6, 8, 10, 12, 14 and 16 and meanwhile, the remaining wound area was measured by analyzing photographs using a digital camera. Images were analyzed with ImageJ software (NIH). The wound closure rate was evaluated. Blood glucose values and weights were also monitored.

#### 2.6.2. Histological Analysis

On days 8 and 14, wounds covered with dressings were sectioned, fixed in 10% (*w*/*v*) neutral buffered formalin, dehydrated through an ethanol series and embedded in paraffin. The embedded specimen of 5~10 µm in thickness were stained with hematoxylin and eosin (H&E) to visualize nuclei or stained with Masson’s trichrome (MT) for collagen or stained with an anti-cluster of differentiation 31 (CD31) antibody (ab182981, Abcam, Cambridge, UK) to observe the growth status of microvascular tissues. All samples were evaluated under optical microscopy (BX41, Olympus, Tokyo, Japan).

## 3. Results and Discussion

### 3.1. Evaluation of the Decellularization Efficiency

**Figure 1:**
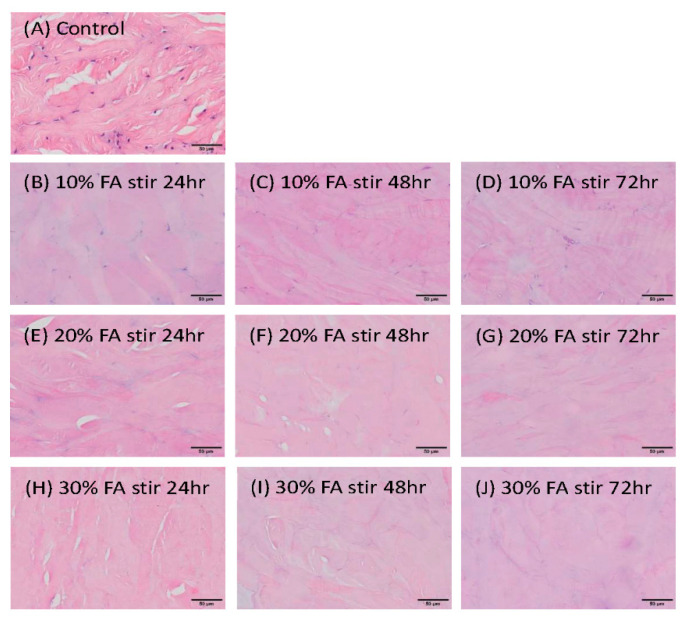
Histological evaluation of porcine skin before (control, **A**) and after decellularization treated with different concentrations of formic acid (FA) (10%, **B**, **C**, **D**; 20%, **E**, **G**, **F**; and 30%, **H**, **I**, **J**) for 24h, **B**, **E**, **H**; 48h, **C**, **F**, **I**; and 72h, **D**, **G**, **J**. Scale bar: 50 μm at 200×.

In our preliminary studies showed that citric acid and malic acid treatments of porcine skin were insufficient and not ideal decellularization reagents to obtain ECM (data not shown). Furthermore, the extent of decellularization of porcine skin treated with formic acid in different concentrations and treatment times was evaluated. Results of the histological evaluation of porcine skin before and after decellularization with H&E staining are shown in Figure 1. As shown by Figure 1A, fresh porcine skin treated with PBS as the control retained nuclei with obvious bluish-purple staining. Those porcine skins treated with 30% formic acid (Figure 1H–J) produced more obvious decellularization effects than that with 10% (Figure 1B–D) and 20% formic acid (Figure 1E–G). Moreover, porcine skin was completely decellularized after continuous treatment with 30% formic acid solution for 72 h. No presence of nuclei (bluish-purple color) or cellular remnants and a looser structure of collagen fibers were observed in decellularized porcine skin treated with 30% formic acid for 72 h (Figure 1J).

The amount of residual DNA, total collagen and GAG of porcine skin following decellularization treatments at various concentrations of formic acid for different times were measured and then compared to fresh porcine skin treated with PBS. These results are listed in Table 3. The DNA content was 349.77 ± 8.33 ng/mg dry tissue weight in porcine skin treated with PBS. DNA quantification showed a rapid decrease in the DNA amount for decellularized skin treated with formic acid and 10% and 20% formic acid had limited decellularization effects on porcine skin, as the content of residual DNA was in the range of 77~93 ng/mg dry weight. Treating porcine skin with 30% formic acid in the first 48 h decreased the DNA content to 58.87 ± 0.51 ng/mg. However, porcine skin immersed in a 30% formic acid solution exhibited decreased amounts of DNA to 67.01 ± 3.75 (19.16% ± 1.07%), 58.87 ± 0.51 (16.83% ± 0.15%) and 42.95 ± 0.73 (12.28% ± 0.21%) ng/mg dry tissue weight after being treated for 24, 48 and 72 h, respectively. Higher concentrations of formic acid solutions (30%) and longer processing times (72 h) were preferred to achieve the best decellularization effect. Prolonged treatment with formic acid, on the other hand, resulted in a compromised ECM. Our studies found that the amount of DNA remaining was 42.95 ± 0.73 ng/mg in dry porcine skin treated with 30% formic acid for 72 h. This meets the criteria for acceptable amounts of residual DNA after decellularization of <50 ng DNA per mg dry weight. Amounts of >75% total collagen still remained after decellularization with various concentrations of formic acid for different time intervals and there was no significant difference compared to the PBS group. These results showed the preservation of collagen, which is one of the major ECM components required for regeneration of skin after decellularization with different treatments in this study. The GAG content of decellularized porcine skin was significantly lower than that in the PBS group, it still remained at 5.50 ± 0.16 µg/mg dry weight (36.96% ± 1.08%) in the treatment with 30% formic acid solution for 72 h. In the 72 h treatment with 30% formic acid, complete decellularization with no presence of nuclei of cellular remnants was observed and the structural pattern of collagen fibers still remained. Although a significant loss of GAG was observed, about 37% of GAG was still retained. Therefore, 30% formic acid treatment for 72 h effectively decellularized porcine skin while retaining the greatest proportion of the ECM. The mechanism of action of formic acid on decellularization is likely due to its high permeability through porcine skin to cause swelling and DNA hydrolysis [32]. The present decellularization method of porcine skin with 30% formic acid treatment for 72 h was easy and efficient without surfactant treatment required, enabling a cost-effective and reproducible process compared to other decellularization procedures previously reported [33,34]. Further, under such a harsh condition at 30% formic acid treated for 72 h, it was expectable that bacteria and virus contamination in the porcine skin should be completely eliminated making *a*ECM a safer biomaterial for preparation of hydrogel dressings.

### 3.2. Preparation and Characterization of aECM Hydrogels

Acellular ECM (*a*ECM) obtained from porcine skin treated with 30% formic acid for 72 h was utilized to prepare *a*ECM hydrogel. It was subjected to digest with pepsin in an acidic solution followed by neutralizing to pH value of 7 and increasing temperature to 37 °C for hydrogel formation. Effects of different concentrations of *a*ECM digested with pepsin at 1 mg/mL concentration in different acidic solution (0.1 N HCl, GA and PCA) on the sol-gel status of hydrogels at 25 and 37 °C are shown in Table 1. At both 25 and 37 °C, formulations of *a*ECM_HCl,10_, *a*ECM_GA,10_ and *a*ECM_PCA,10_ were observed to be in solution form, whereas that for *a*ECM_HCl,50_, *a*ECM_GA,50_ and *a*ECM_PCA,50_ were in gel form. Favorably, formulations of *a*ECM_HCl,25_, *a*ECM_GA,25_ and *a*ECM_PCA,25_ were observed to be in sol form at 25 °C, then transformed into a gel or glutinous form at 37 °C. This indicated that a concentration of at least 25 mg/mL of *a*ECM was needed in order to assembly pepsin-soluble ECM fibers into 3-dimentional hydrogel structure after neutralization to a pH value of 7 and increasing temperature to 37 °C. Further, the digestion efficiency of pepsin seems to be more appropriate in 0.1 N HCl than in GA or PCA solution.

**Figure 2:**
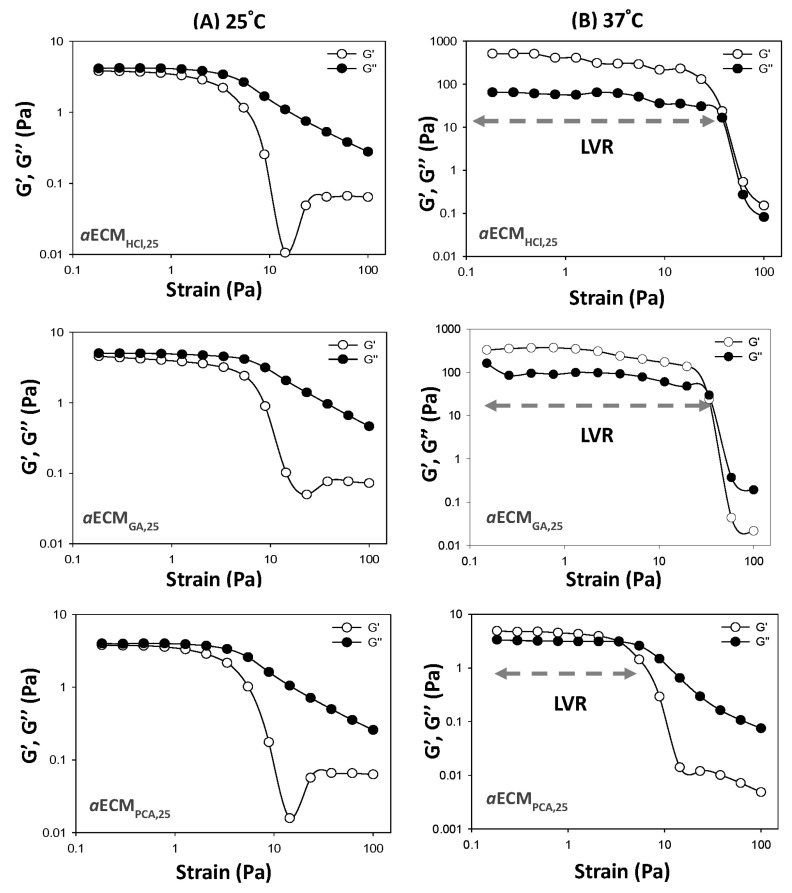
Rheological analysis of *a*ECM_HCl,25_, *a*ECM_GA,25_ and *a*ECM_PCA,25_ at 25 (**A**) and 37 °C (**B**).

Rheological behaviors of *a*ECM_HCl,25_, *a*ECM_GA,25_ and *a*ECM_PCA,25_ hydrogels were further evaluated at 25 and 37 °C and results are shown in Figure 2. Rheological profiles for *a*ECM_HCl,25_, *a*ECM_GA,25_ and *a*ECM_PCA,25_ hydrogels (Figure 2A) showed to be in a sol form at 25 °C as indicated by G″ (viscous modulus) > G′ (elastic modulus), while they all transformed into a gel form at 37 °C as indicated by G′ > G″ (Figure 2B). The length of the linear viscoelastic region (LVR) of hydrogels determines the hydrogel structural stability that is a measure of hydrogel resistance to strain force. Those hydrogels with a longer length of LVR should possess a higher hydrogel strength which is only able to be destructed at a higher strain force, indicating that hydrogels could be retained firmly on the wound site. As shown by Figure 2B, the order of LVRs was as followed—*a*ECM_HCl,25_ > *a*ECM_GA,25_ >> *a*ECM_PCA,25_. These results demonstrated that *a*ECM_HCl,25_ hydrogel was optimal with a sol form at 25 °C for easier application on the wound area and transforming into a gel form at 37 °C with a gel strength higher enough for firmly retaining. Pepsin-soluble collagen (PSC) hydrogel with similar rheological behaviors has been reported by Ge et al. [15]. But it did not demonstrate that there exists a sol-gel transition characteristic at 37 °C for PSC hydrogel.

**Figure 3:**
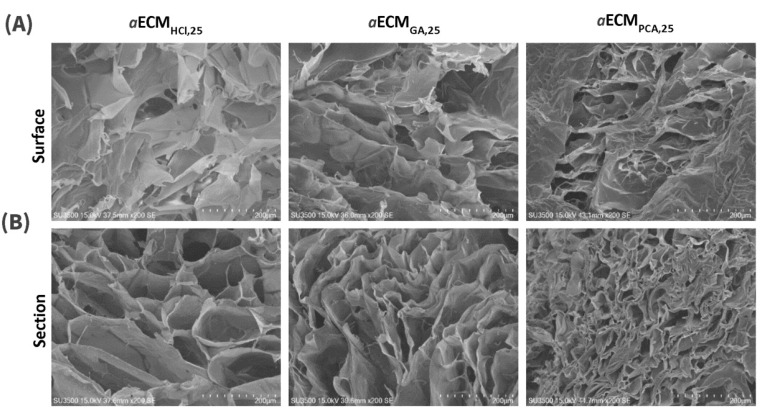
Scanning electron microscopy (SEM) images ((**A**) surface and (**B**) cross-section, scale bar: 200 µm) of *a*ECM_HCl,25_, *a*ECM_GA,25_ and *a*ECM_PCA,25._

**Figure 4:**
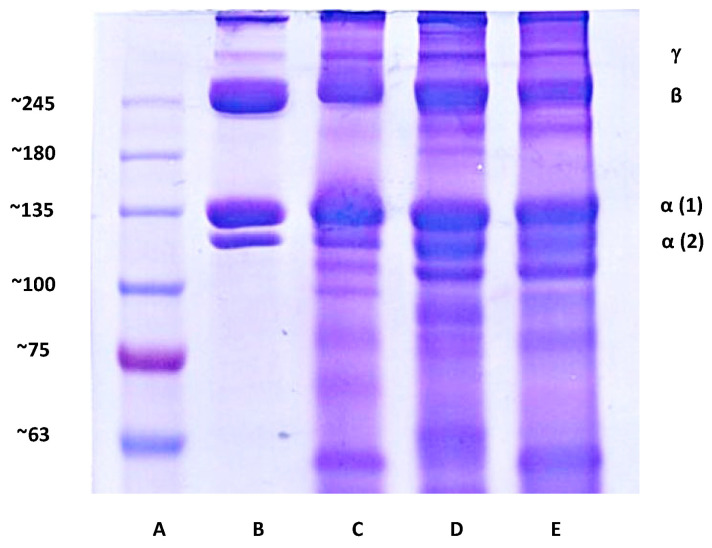
Sodium dodecylsulfate-polyacrylamide gel electrophoresis (SDS-PAGE) electrophoresis patterns. (**A**) Marker, (**B**) porcine type I collagen standard, (**C**) *a*ECM_HCl,25_ hydrogel, (**D**) *a*ECM_GA,25_ hydrogel and (**E**) *a*ECM_PCA,25_ hydrogel.

In the SEM morphological examination as shown in Figure 3, the surfaces of *a*ECM_HCl,25_, *a*ECM_GA,25_ and *a*ECM_PCA,25_ all showed similar flaky structures (Figure 3A). Meanwhile, they all showed highly porous structures on cross-section but *a*ECM_HCl__,25_ possessed larger pore sizes compared to the others (Figure 3B). Figure 4 shows the SDS-PAGE electrophoretic patterns of the porcine type I collagen standard, *a*ECM_HCl,25_, *a*ECM_GA,25_ and *a*ECM_PCA,25_. All three hydrogels of *a*ECM_HCl,25_, *a*ECM_GA,25_ and *a*ECM_PCA,25_ displayed similar α1, α2, β and γ four bands as the collagen standard and the content of α1 was about twice that of α2, which was consistent with the molecular composition of type I collagen (α1)_2_α2. This seems to reveal that the structural patterns of collagen fibers still remained in the *a*ECM_HCl,25_, *a*ECM_GA,25_ and *a*ECM_PCA,25_ samples. Overall *a*ECM_HCl,25_ hydrogel was confirmed to be optimal for utilization as wound dressing with minimal loss of extracellular matrix and less modification of collagen fibers.

### 3.3. In Vitro Cell Viability Test

**Figure 5:**
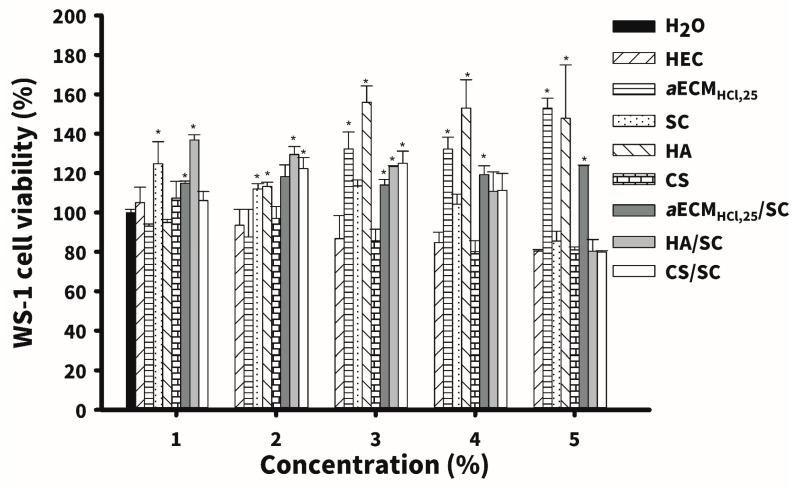
Cell viability (*n* = 3) of WS-1 human skin fibroblasts incubated with five kinds of hydrogel dressings including hydroxyethyl cellulose (HEC), sacchachitin (SC), chitosan (CS) and hyaluronic acid (HA and *a*ECM_HCl,25_ and three kinds of composite hydrogel including *a*ECM_HCl,25_/SC, HA/SC and CS/SC at a concentration of 0.005%, 0.01%, 0.05%, 0.1% and 0.5% for 72 h. Values are presented as the mean ± standard deviation. * *p* < 0.05, compared to H_2_O.

Five kinds of hydrogel dressings including HEC, CS, SC, HA and *a*ECM_HCl,25_ and three kinds of composite hydrogel including *a*ECM_HCl,25_/SC, HA/SC and CS/SC were selected to conduct regenerative healing studies on diabetic wounds. In vitro studies of cell viability first examined for these hydrogel dressings. After 72 h of culture, results of cell viability of WS-1 human fibroblasts with different concentrations of these hydrogel dressings are shown in Figure 5. Results showed that no hydrogel dressings possessed any toxicity at all concentrations examined (WS-1 cell viability >80%). Moreover *a*ECM_HCl,25_, HA and *a*ECM_HCl,25_/SC at higher concentrations (>0.05%) caused statistically significant proliferation of WS-1 cells compared to two control groups of H_2_O and HEC (*p* ≤ 0.05). It reveals that the enhancement of proliferation of WS-1 human fibroblasts by *a*ECM_HCl,25_ and *a*ECM_HCl,25_/SC is conformed to that as reported previously [27,35]. Promotion of fibroblast proliferation by HA hydrogel is consistent with that reported by Ciccone et al. [36]. It further confirmed that these three hydrogel dressings were very suitable for diabetic wound application.

### 3.4. In Vivo Wound-Healing Studies

**Figure 6:**
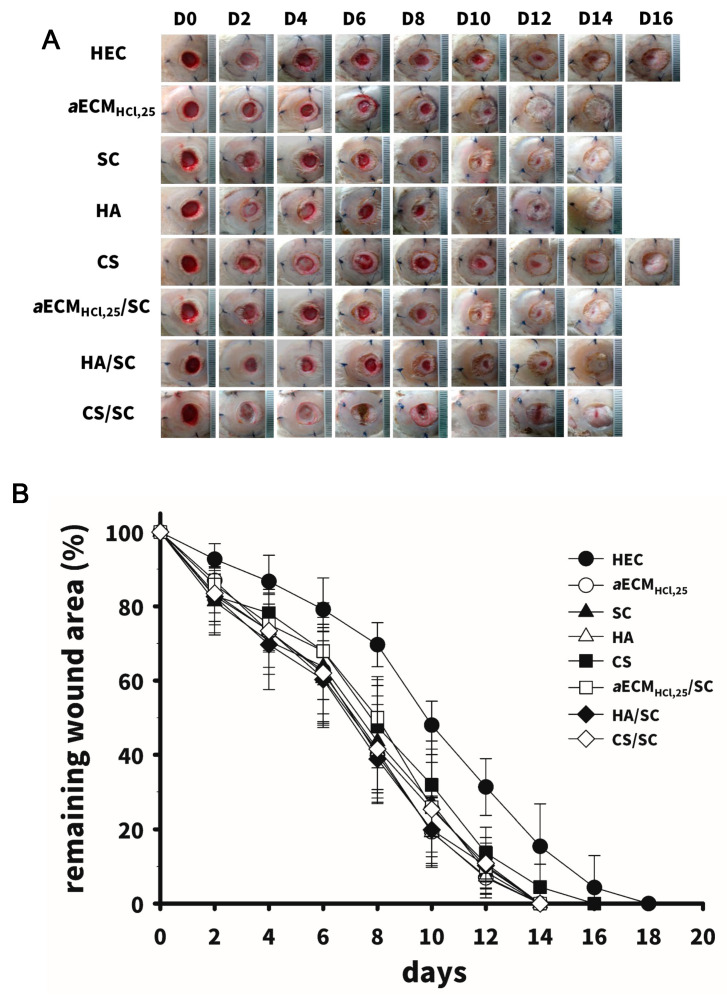
Wound-healing studies (*n* = 4~6) in diabetic rats showing the patterns of the healing process vs. time (**A**) with five kinds of hydrogel dressings including HEC, CS, SC, HA and *a*ECM_HCl,25_ and three kinds of composite hydrogel including *a*ECM_HCl,25_/SC, HA/SC and CS/SC (C); (**B**) The remaining wound area vs. time profiles. Values are presented as the mean ± standard deviation. * *p* < 0.05, compared to H_2_O.

An in vivo study of wound healing was conducted on a diabetic wound model of rats with streptozocin-induced diabetes. Photo images of wounds treated with the above-described eight types of hydrogel dressings are depicted in Figure 6A and quantitative measurements of the wound area are shown in Figure 6B. The wound lesions had completely healed after 16 days of covering with any of the wound dressings. A thin epithelial layer completely sealing the wounds was clearly visible in wounds for the *a*ECM_HCl,25_, SC, *a*ECM_HCl,25_/SC and HA/SC groups on day 14. A rapid drop in the remaining wound area on days 8 to 12 was observed. A significantly smaller remaining wound area was observed in the *a*ECM_HCl,25_, SC, *a*ECM_HCl,25_/SC and HA/SC groups compared to the HEC group after day 10 (*p* < 0.05) and wound lesions had completely sealed by day 14.

**Figure 7:**
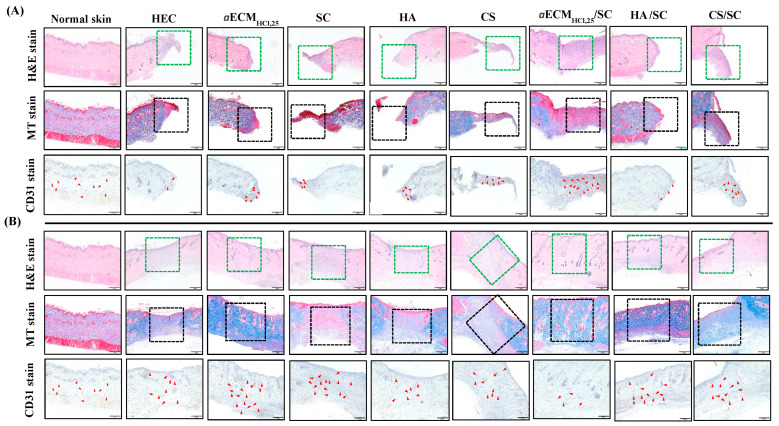
Histologic analysis of normal skin and wounds treated with different dressings. Top to bottom displays hematoxylin and eosin (H&E), Masson’s trichrome (MT) and cluster of differentiation 31 (CD31) immunohistochemical staining. (**A**) Day 8, (**B**) day 14. Scale bar: 500 µm at 4×.

Histological examination of the entire wound healing process for all hydrogel dressings was performed and compared to normal skin on days 8 and 14 (Figure 7). The histological evaluation with H&E staining showed that all groups still presented wounded skin on day 8 and they showed a purple stain in the wounded areas within the green dashed lines. Newly grown tissue around the wound was observed with all dressings. The wound was in the last stage of inflammation (Figure 7A top). On day 14, the wound had closed and no inflammation was evident (Figure 7B top). A moderate number of fibroblasts occupied the dermis, which was considered remodeling in all groups. However, wounds treated with *a*ECM_HCl,25_, *a*ECM_HCl,25_/SC and HA/SC had healed as functional tissue with generation of hair follicles and sweat glands and exhibited complete skin regeneration. To further evaluate the wound-healing response, MT stain was used to examine the deposition of collagen in the wounds. On day 8, all groups had less collagen production with loose of structure indicating the early stage of remodeling as shown in areas within the black dashed lines (Figure 7A middle). The collagen had grown messily with no regular arrangement. Results showed that inflammation was still undergoing and the dermis demonstrated incomplete remodeling on day 8. In the *a*ECM_HCl,25_, *a*ECM_HCl,25_/SC and HA/SC groups, collagen had completely occupied the dermis with a uniform distribution of collagen fibers by day 14 and thus they had the most complete remodeling outcomes. Favorably, the *a*ECM_HCl,25_/SC group demonstrated to have similar histologic images as that for the normal skin with a short wound-healing duration (Figure 7B middle). CD31 staining was used to evaluate vascularization in each experimental group on days 8 and 14 and results are also shown in Figure 7. As can be seen, large numbers of micro-blood vessels (red arrow) were observed in the *a*ECM_HCl,25_/SC group compared to the other groups on day 8 and the wounded tissue had greater blood flow to provide sufficient oxygen and nutrition to the tissues (Figure 7A bottom). This indicated that wounds treated with *a*ECM_HCl,25_/SC had proceeded to the remodeling phase by facilitating neovascularization to accelerate wound healing on day 8. However, the other groups had begun to exhibit greater vascularization only by day 14 with delayed wound healing while the *a*ECM_HCl,25_/SC group exhibited the least vascularization and had full wound healing by day 14 (Figure 7B bottom). Overall, wound healing treated with various dressings was scored on the basis of scarring and H&E, MT and CD31 staining on day 14 and results were shown in Table 4. The degree of wound healing was graded from 1 to 5 depending on the extent of remodeling and the total score of normal skin was 20. It was observed for wounds treated with *a*ECM_HCl,25_/SC after 14 days that the degree of remodeling in all items including scarring and H&E, MT and CD31 staining was graded as the highest among these hydrogel dressings examined. All of the above results showed that *a*ECM_HCl,25_/SC had the potential to promote diabetic wound healing.

The mechanism of accelerating wound healing by the ECM_HCl,25_/SC hydrogels is rapid re-epithelialization and normal ECM deposition due to enhanced cellular movements in the moist microenvironment. Many studies also showed that ECM hydrogels promote cell infiltration, particularly by macrophages and progenitor cells, neovascularization and positive functional remodeling [20]. Further, *a*ECM_HCl,25_/SC hydrogels accelerated wound healing to a greater extent than that for each of individual hydrogel dressings (*a*ECM_HCl,25_ and SC). It indicated that there exists a potential of synergistic promotion of diabetic wound healing. The underlying mechanism responsible for that might be attributed that SC was able to promote wound healing as a result of its chemotactic effect on inflammatory cells, in turn, facilitating subsequent angiogenesis, granulation tissue formation and more-rapid new tissue formation, leading to faster wound healing [26,32]. Combination therapies with a composite hydrogel composed of *a*ECM_HCl,25_ and SC hydrogel may improve the therapeutic effects of *a*ECM hydrogels in diabetic wounds. A diabetic wound covered by an *a*ECM_HCl,25_/SC hydrogel had completely healed by 14 days in the current study, whereas Hung et al. reported that skin wounds covered with an SC membrane needed 21 days [35]. It might be attributed to synergistical promotion of wound healing with a composite hydrogel. Furthermore, in comparison to SC membrane, *a*ECM_HCl,25_/SC hydrogels are designed to hydrate wounds, thereby providing an ideal environment for wound healing. With their high moisture content (>90% water), they help prevent bacteria and oxygen from reaching the wound, providing a barrier against infection.

**Figure 8:**
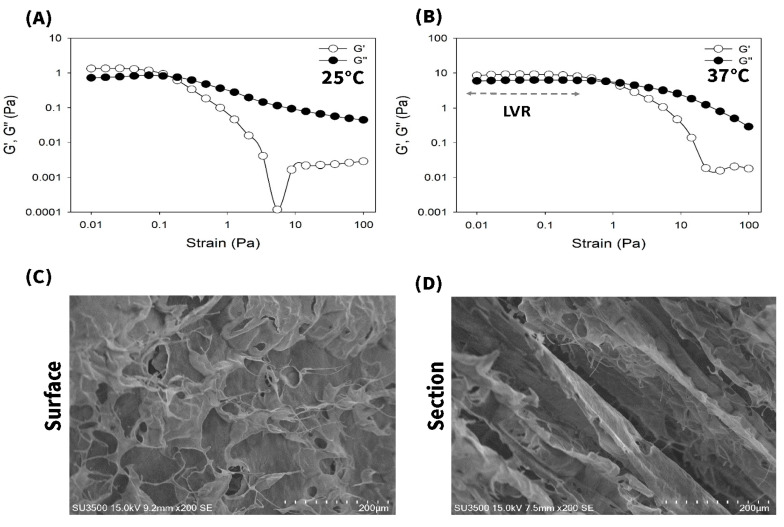
Rheological analysis of *a*ECM_HCl,25_/SC wound dressing at (**A**) 25 and (**B**) 37 °C. SEM images of *a*ECM_HCl,25_/SC sponge on the surface (**C**) and the cross-section (**D**) (scale bar: 200 µm).

Finally, the rheological behaviors and SEM morphological image of *a*ECM_HCl,25_/SC hydrogel were examined and results exhibited in Figure 8. Results reconfirmed that *a*ECM_HCl,25_/SC was still the same as *a*ECM_HCl,25_ in sol form at 25 °C for easier application on the wound area and was able to transform into a gel form at 37 °C with a gel strength still higher enough for firmly retaining (Figure 8A,B). Further, this composite hydrogel dressing of *a*ECM_HCl,25_/SC was observed to be non-adhesive to the wound and thus can be easily removed without causing any trauma or pain. Accordingly, *a*ECM_HCl,25_/SC hydrogels can potentially be utilized for accelerating diabetic wound repair and skin regeneration with all key requirements of a material that facilitates the healing process of the wound.

In comparison with wound healing effects of paste type of acellular dermal matrix subcutaneous injection reported by Lee et al. [37], it was only able to achieve 40% change in wound size for 14-day treatment of full-thickness skin defect indicating that *a*ECM_HCl,25_/SC as a hydrogel form might be more beneficial to diabetic wound healing than that in paste type. However, Jeon and Kim reported that CGPaste (a paste type of acellular dermal matrix) was an effective option for coverage of small and deep chronic wounds (the mean wound area was 453.57 mm^2^ and the depth was 10.71 mm) by demonstrating that the wound healing occurred in five of the seven patients (71.43%) with the mean duration of complete healing being 2.4 weeks, which was similar to that for *a*ECMHCl,25/SC as a hydrogel form [19]. Huang et al. [38] reported that after 21 days of wound healing, 80% and 84% of the wound area had re-epithelialized for symmetric Trp-rich peptides (PSI)-loaded poly (ethylene glycol) diacrylate (PEGDA)/chitosan (CS) hydrogel and PSI-plasmid Ang-1 (pANG)-loaded PEGDA/thiolated chitosan (TCS) hydrogel group, whereas only 60% and 70% of the wound area had healed for the control and PEGDA group in the meantime demonstrating that *a*ECM_HCl,25_/SC as a hydrogel form illustrates better wound healing promotion efficacy as well. Lee et al. [39] reported that after applying a paste formulation of acellular dermal matrix (ADM) to the Diabetic foot ulcers (DFUs) resulted in the mean times to heal (within 60 days) of 13.54 ± 9.18 days and 21.5 ± 11.98 days, respectively, for the treatment group and the control group showing that the paste formulation of ADM equivalently provides a matrix for tissue ingrowth and promotes the healing of diabetic wound healing to that for *a*ECM_HCl,25_/SC as a hydrogel form. Further, Griffin et al. [40] reported that the median time to 75% to 100% granulation being 42 days for the oxidized regenerated cellulose (ORC)/collagen/silver-ORC dressing group versus 60 days for the bovine collagen extracellular matrix (ECM), concluding that *a*ECM_HCl,25_/SC as a hydrogel form might be expectable to be more favorable to diabetic wound healing.

## 4. Conclusions

In conclusion, a simple and effective decellularization method by treating porcine skin with 30% formic acid for 72 h was successfully developed to obtain *a*ECM with retaining >75% collagen and ~37% GAG with the presence of the nuclei of cellular remnants to an extent of below minimal acceptable amount. The pepsin-soluble *a*ECM_HCl,25_ hydrogel could be fabricated at as a minimum of 25 mg/mL concentration of so-obtained *a*ECM digesting with 1 mg/mL of pepsin in 0.1 N HCl. *a*ECM_HCl,25_ hydrogel exhibited a porous morphology with no toxicity as a biomaterial with optimal characteristics for promoting wound healing. Furthermore, a composite hydrogel consisting of *a*ECM_HCl,25_ and SC (*a*ECM_HCl,25_/SC) showed to synergistically accelerate wound healing and provided the healed wound with functional tissues in diabetic wound-healing studies. Ultimately, the same decellularization method to obtain *a*ECM and the fabrication procedure of *a*ECM hydrogel potentially play as a technical platform, on which *a*ECM hydrogels could be created from numerous tissue sources in almost every organ system. A composite hydrogel combined with sacchachitin can then be applied to facilitate functional and constructive tissue remodeling in a variety of clinical applications in the future.

## Figures and Tables

**Table 1:** Effects of different concentrations of acellular extracellular matrix (*a*ECM) digested with pepsin in different acidic solution on the sol-gel status of hydrogels at 25 and 37 °C.

Acronym	*a*ECM (mg/mL)	Acid	25 °C	37 °C
*a*ECM_HCl,10_	10	0.1 N HCl	Solution	Solution
*a*ECM_GA,10_	10	GA	Solution	Solution
*a*ECM_PCA,10_	10	PCA	Solution	Solution
*a*ECM_HCl,25_	25	0.1 N HCl	Solution	Gel
*a*ECM_GA,25_	25	GA	Solution	Glu
*a*ECM_PCA,25_	25	PCA	Solution	Glu
*a*ECM_HCl,50_	50	0.1 N HCl	Gel	Gel
*a*ECM_GA,50_	50	GA	Gel	Gel
*a*ECM_PCA,50_	50	PCA	Gel	Gel

GA, glycolic acid; PCA, 2-pyrrolidone-5-carboxylic acid; Glu, Glutinous solution.

**Table 2:** Formulations of hydrogel dressings.

Hydrogel Dressings	Formulations
HEC	300 mg HEC in 20 mL ddH_2_O
*a*ECM_HCl,25_	500 mg *a*ECM_HCl,25_ in 20 mL 0.1 N HCl
SC	400 mg SC in 20 mL ddH_2_O
HA	400 mg HA in 20 mL ddH_2_O
CS	400 mg CS in 20 mL 0.1 N glycolic acid adjusted to pH 7.0 with 2 N NaOH
*a*ECM_HCl,25_/SC	10 mL *a*ECM_HCl,25_ + 10 mL SC
HA/SC	200 mg HA in 20 mL SC
CS/SC	400 mg CS in 20 mL SC

HEC, hydroxyethyl cellulose; SC, sacchachitin; HA, hyaluronic acid; CS, chitosan.

**Table 3:** Contents of residual DNA, total collagen and glycosaminoglycan (GAG) of porcine skin following decellularization at various concentrations of formic acid (FA) for different time points compared to fresh porcine skin treated with phosphate-buffered saline (PBS) under the same processing steps. Values are presented as the mean ± standard deviation (*n* = 3). (* *p* < 0.05 compared to fresh porcine skin treated with PBS).

Treatments	0 h	24 h	48 h	72 h
Residual DNA (ng/mg dry weight)				
PBS	349.77 ± 8.33	-	-	-
10% FA	-	93.43 ± 1.14 *	90.89 ± 2.02 *	87.16 ± 2.86 *
20% FA	-	87.84 ± 0.85 *	87.35 ± 3.90 *	77.32 ± 0.82 *
30% FA	-	67.01 ± 3.75 *	58.87 ± 0.51 *	42.95 ± 0.73 *
Total collagen (µg/mg dry weight)				
PBS	715.39 ± 7.34	-	-	-
10% FA	-	647.15 ± 18.51	630.45 ± 13.43	625.87 ± 2.38
20% FA	-	610.25 ± 2.13	583.85 ± 3.90	566.52 ± 7.34
30% FA	-	574.69 ± 7.46	567.24 ± 7.97	556.01 ± 5.94
Glycosaminoglycan (µg/mg dry weight)				
PBS	14.88 ± 1.21	-	-	-
10% FA	-	10.61 ± 0.32 *	8.97 ± 0.40 *	9.59 ± 0.32 *
20% FA	-	9.19 ± 0.08 *	7.79 ± 0.08 *	6.52 ± 0.32 *
30% FA	-	7.09 ± 0.64 *	6.86 ± 0.80 *	5.50 ± 0.16 *

**Table 4:** Overall assessment for in vivo wound healing studies on day 14.

Wound Dressings	Scarring	H&E	MT	CD31	Sum of Grading Score
Normal skin	5	5	5	5	20
HEC	1	1	1	3	6
ECM_HCl_	4	3	3	4	14
SC	4	1	1	3	9
HA	3	1	1	3	8
CS	1	1	1	3	6
ECM_HCl_+SC	4	4	5	4	17
HA+SC	4	2	3	3	12
CS+SC	2	1	1	3	7

Note: The degree of wound healing was graded from 1 to 5 depending on the extent of remodeling: 1 = minimal (1%~20%); 2 = slight (21%~40%); 3 = moderate (41%~60%); 4 = moderately high (61%~80%); 5 = high (81%~100%). H&E, hematoxylin and eosin; MT, Masson’s trichrome; CD31, cluster of differentiation 31.
